# Pathos: A web facility that uses metabolic maps to display experimental changes in metabolites identified by mass spectrometry

**DOI:** 10.1002/rcm.5245

**Published:** 2011-10-17

**Authors:** David P Leader, Karl Burgess, Darren Creek, Michael P Barrett

**Affiliations:** 1 School of Life Sciences, College of Medical, Veterinary and Life Sciences, University of GlasgowGlasgow, G12 8QQ, UK; 2 Functional Genomics and Systems Medicine, College of Medical, Veterinary and Life Sciences, Joseph Black Building, University of GlasgowGlasgow, G12 8QQ, UK; 3 Wellcome Trust Centre for Molecular Parasitology, Institute of Infection, Immunity and Inflammation, College of Medical, Veterinary and Life Sciences, University of GlasgowGlasgow, G12 8TA, UK; 4 Department of Biochemistry and Molecular Biology, Bio21 Molecular Science and Biotechnology Institute, University of MelbourneFlemington Rd, Parkville, Victoria, 3010, Australia

## Abstract

This work describes a freely available web-based facility which can be used to analyse raw or processed mass spectrometric data from metabolomics experiments and display the metabolites identified – and changes in their experimental abundance – in the context of the metabolic pathways in which they occur. The facility, *Pathos* (http://motif.gla.ac.uk/Pathos/), employs Java servlets and is underpinned by a relational database populated from the Kyoto Encyclopaedia of Genes and Genomes (*KEGG*). Input files can contain either raw *m/z* values from experiments conducted in different modes, or *KEGG* or *MetaCyc* IDs assigned by the user on the basis of the *m/z* values and other criteria. The textual output lists the *KEGG* pathways on an XHTML page according to the number of metabolites or potential metabolites that they contain. Filtering by organism is also available. For metabolic pathways of interest, the user is able to retrieve a pathway map with identified metabolites highlighted. A particular feature of *Pathos* is its ability to process relative quantification data for metabolites identified under different experimental conditions, and to present this in an easily comprehensible manner. Results are colour-coded according to the degree of experimental change, and bar charts of the results can be generated interactively from either the text listings or the pathway maps. The visual presentation of the output from *Pathos* is designed to allow the rapid identification of metabolic areas of potential interest, after which particular results may be examined in detail. Copyright © 2011 John Wiley & Sons, Ltd.

The application of high-resolution mass spectrometry to the analysis of the abundance of small molecules^1^ has allowed large numbers of cellular metabolites to be identified simultaneously and changes in their concentrations to be studied under different experimental conditions. Raw data from mass spectrometry typically contain both noise and a large number of genuine metabolite peaks, and sophisticated methods have been developed for refining these data and determining the possible molecular formulae corresponding to each detected molecular mass (reviewed in Castillo *et al*.^2^). The first stages of such refinement typically involve peak detection, filtering, grouping, and sample alignment.^3^,^4^ Exact molecular mass alone does not generally allow unequivocal identification of metabolites because of the existence of isomers, so this stage may be followed by attempts at identification and annotation on the basis of a variety of criteria,^5^–^7^ using MS/MS fragmentation or chromatographic information,^8^ and considering the results in the context of metabolic pathways or networks.^9^ Applications exist to integrate these different stages into a single tool or pipeline.^7^,^10^

A final stage in this pipeline is integrating identified compounds into a visual metabolic context, and there are two broad approaches to this. One is to generate network diagrams.^11^–^13^ Although this can be very powerful, biologists often wish to view metabolites in the context of a representation of metabolic pathways with which they are more familiar. *KEGG* has developed a *Mapper* tool^14^ that enables interactive imposition and colour highlighting of metabolites in their pathway maps, and this interactive approach has been extended further in *iPath*.^15^ However, these facilities are not designed for routine analysis of mass spectrometry data. Bespoke facilities^16^,^17^ for visualizing metabolites from mass spectrometry have been provided for the different representations offered by the two main databases of metabolic pathways, *MetaCyc*^18^ and *KEGG*.^19^ The *MassTRIX* facility,^17^ which employs the *KEGG* pathways environment to represent metabolomics data, has been a useful and simple tool with which to contextualize metabolomics data sets. However, in relation to our own work, it lacked an important feature, namely the ability to accept and display comparative data from different experimental conditions. *Pathos*, the metabolomics web facility described here, fulfils this requirement and, in addition, is able to accept identified metabolites as an alternative to *m/z* values with rapid feedback of results, thus expanding the range of data types that can be analyzed.

## EXPERIMENTAL

The *MySQL* relational database underlying *Pathos* contains tables of data for metabolites, for pathway maps and for organisms, and is described in the Supporting Information. The database was populated from files downloaded from the *KEGG* FTP site^20^ after processing with parsers written in Java for the purpose.

The core of the web facility is a Java servlet, the source code for which may be downloaded from the internet^21^ and used under GPL license conditions. The servlet loads the data for the user-specified organism (or all organisms) from the database and holds it in memory in the form of objects representing pathway maps. Metabolites or potential metabolites derived from the user's uploaded input file are added to these pathway objects. If the input file lists *KEGG* or *MetaCyc* IDs this is done directly. If the input file contains *m/z* values from analysis performed in positive or negative ion mode, possible neutral exact masses are calculated (for the most abundant isotope of each atom) by considering each of a number of possible adducts: 32 in the case of positive mode, and 15 in the case of negative mode (listed at^22^). Potential metabolites are then assigned from these neutral masses, according to the degree of mass accuracy selected by the user, and this latter procedure is what occurs if the user uploads *m/z* values in 'neutral mode' (i.e. *m/z* values from positive or negative mode that have been adjusted by pre-processing software, usually assuming that the charged species resulted from the gain or loss, respectively, of a proton). If a metabolite in an experimental data set is identified on the basis of more than one *m/z* value (e.g. from different adducts) only the data containing the highest value are taken and the others discarded.

The metabolite information in the pathway objects is formulated as an XML-processable XHTML page, which is returned to the client. This page has several interactive user options that employ additional server software. Customized images of pathway maps may be generated via a Perl CGI application that makes use of *KEGG* web services, column charts of the results of experiments may be generated from a small bespoke Java servlet, *MSCompare*, and details of metabolites may be generated from a small bespoke Perl CGI application, *formula.cgi*.

As the processing is all performed on the server, the demands that *Pathos* makes on the user's hardware and software are extremely modest. Operating systems as old as Windows XP and Mac OS X 10.4 have been found to be quite adequate with standard web browsers such as *Firefox*, *Safari*, *Chrome* or *Opera* (with JavaScript enabled). Unfortunately, *Microsoft Internet Explorer* presents technical problems that exclude its use. We currently run the public version of the *Pathos* web application on a 2.2 GHz dual-processor machine running Linux, the Sun Java System Web Server 7.0, and *MySQL* server version 5. However, in developmental and on local intranets we have employed desktop and laptop machines with lower configurations: single 1.4 GHz processors, Apache Tomcat 4.1, and *MySQL* server version 4.

## RESULTS

**Figure 1:**
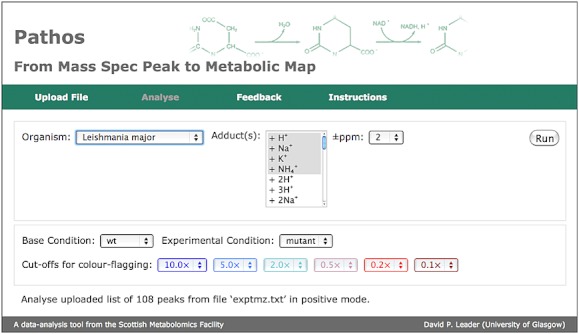
*Pathos*: view of an XHTML page showing options following upload of file *m/z* values from a mass spectrometry analysis performed in positive ion mode for an experiment comparing wild-type *L. mexicana* with a glucose transporter-defective mutant. With the exception of the organism selection, the settings shown are the defaults.

We illustrate the use of *Pathos* with 'exptmz.txt', one of the experimental files that can be downloaded from the home page.^23^ The focus of this description is on the output obtained – instructions, including details of file formats, are available on-line or as a downloadable PDF file. The file 'exptmz.txt' contains *m/z* values from a mass spectrometry analysis (performed in positive ion mode) of wild-type and glucose transporter-defective *Leishmania mexicana*.^24^ On uploading the file from the home page, the Java servlet returns a page of analysis options and confirmation of the number of peaks read (Fig. 1). The user may choose from a list of organisms (or accept the default of 'All Organisms'), from a range of adducts appropriate to the analytical method (a 'base' set is selected by default), and may alter the mass tolerance (in ppm) if desired. To allow colour highlighting of changes in the concentration of metabolites in experiments with multiple conditions, 'base' and 'experimental' conditions are specified in the input file. These specifications are displayed on pull-down lists, but may be changed by the user at any time during a session.

**Figure 2:**
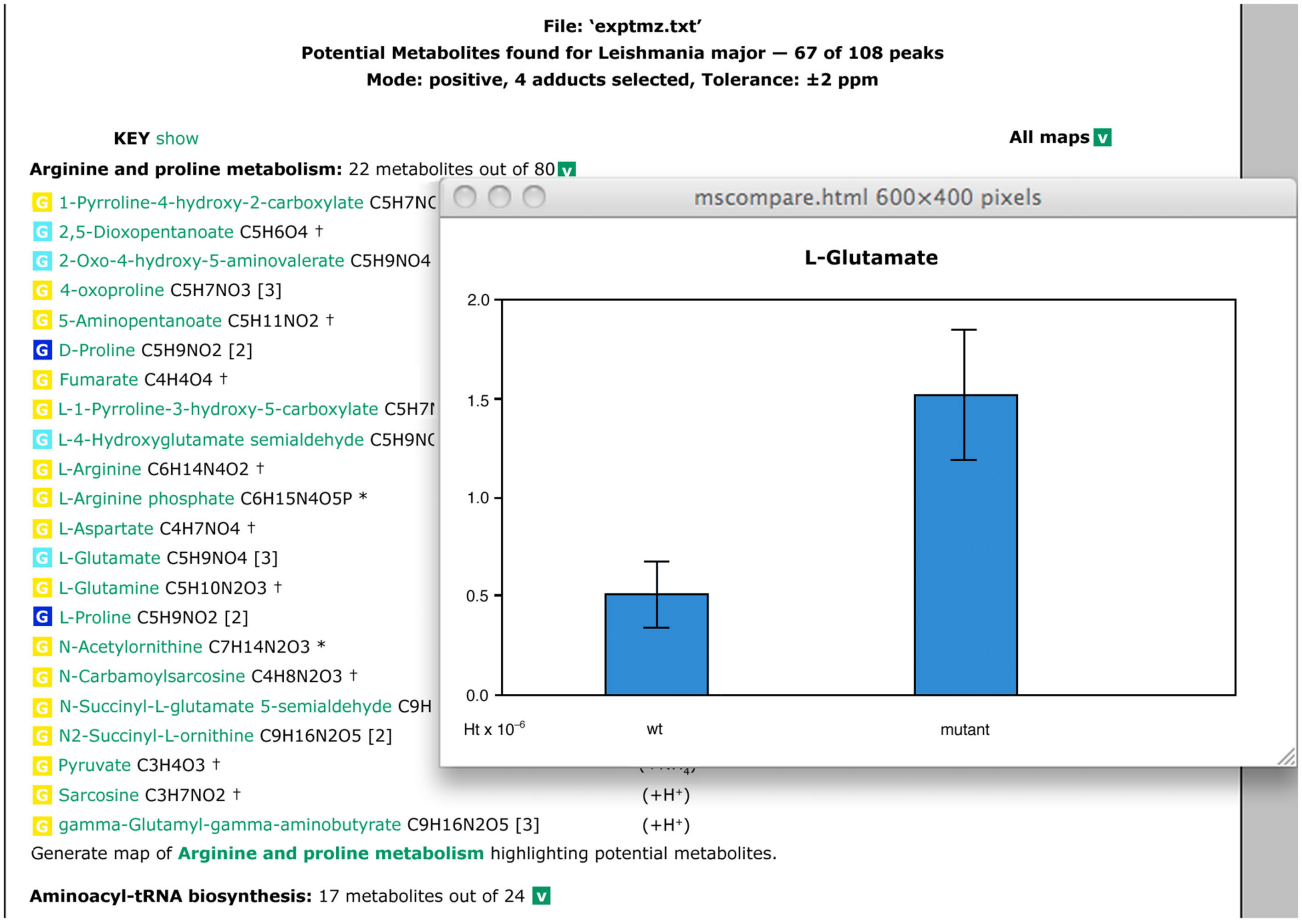
*Pathos*: view of textual output following analysis, and column chart of experimental results for a particular metabolite. For explanation, see text.

After running the analysis a web page is returned with pathway listings initially shown as summaries sorted by the number of identified metabolites in a pathway, allowing a quick overall survey of the results. Individual listings of interest may be expanded to reveal the details of the (putative) metabolites, as is shown for Arginine and Proline metabolism in Fig. 2. The 'G' (graph) symbol to the left of the name of each metabolite is a hyperlink that can be clicked to generate a column chart of the experimental changes for the metabolite (floating pop-up in Fig. 2). The 'G's are colour-coded to indicate the degree of change (dark blue – most positive, dark red – most negative) allowing the user to focus on those metabolites that show the greatest changes with respect to induced perturbation. (There is an option to expand all the listings at once for the user who wishes to scan them for metabolites that have undergone experimental changes.)

It is recognized that identification of metabolites by exact mass (even within a mass accuracy of 1 ppm) is insufficient when all possible molecular formulae are considered;^25^ however, annotation of formulae by exact mass (i.e. from accurate *m/z* data) *is* sufficient for most formulae of known metabolites in the *KEGG* database (only eight overlapping pairs were found within a tolerance of 2 ppm in ca. 2300 metabolites – see Supporting Information). Nevertheless, there are many cases in which formulae correspond to multiple isomers in the database, thus preventing accurate identification of metabolites from *m/z* data. The user is alerted to this latter problem by the pathway listings, as follows. Metabolites, the formulae of which are not represented by isomers in the database (i.e. which are more likely to have been correctly identified), have an asterisk appended, those with isomers that are not present in the current pathway have a dagger appended, and those with isomers that are present in the current pathway have the number of such isomers appended in parentheses (visible in Fig. 2). Furthermore, if the cursor is held over a formula, all instances of it are highlighted. A facility is also provided whereby clicking on the name of a metabolite generates a small pop-up window containing a complete list of isomers of the same mass in the database. (This window also presents the molecular structure of the metabolite – not illustrated, but see Supporting Information.)

In the case of positive or negative mode *m/z* data, the adduct for the most intense peak is indicated in parentheses. At the foot of the page there is a listing of the molecular formulae corresponding to each of the identified peaks, with the adduct indicated, whether or not the peak was used in the pathway sections. Listings of unidentified peaks and of pathways lacking metabolites are also provided.

**Figure 3:**
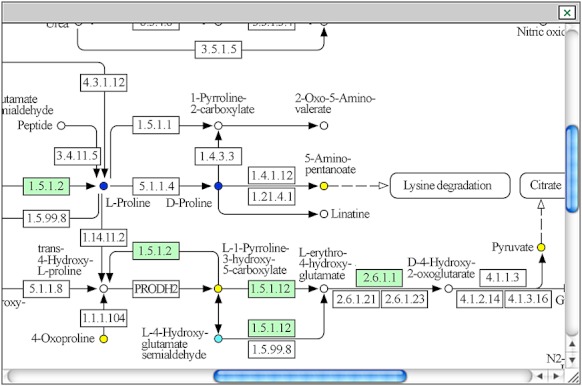
*Pathos*: metabolic pathway map. The map window has been decreased in size for convenience, and the visible portion chosen to illustrate a clustering of identified metabolites, some of which had increased in concentration in the mutant (blue circles). The light-green background for the EC numbers of particular enzymes indicates that they are predicted to be present in the genome of *Leishmania major* (a parasite closely related to *L. mexicana*).

Each pathway is furnished with an option that allows the user to generate an annotated map through a call to the *KEGG* web service. (Generation of the pathway map by the *KEGG* web services takes 15 s or longer, so that a temporary 'busy' icon is provided in the place where the map would appear to reassure the user that the call has not failed.) A portion of such a map is shown in Fig. 3, where it can be seen that those circles representing identified metabolites are colour-coded in the same manner as the 'G's, above. They are also hyperlinks, and clicking them invokes the corresponding column chart. The map and column-chart graphics are PNG images which can be saved to disc.

If the user provides a file containing either *KEGG* or *MetaCyc* IDs, rather than *m/z* values, operation and results are similar, but an appropriately simpler interface and results listing are provided. If the data are simply in a single list of *m/z* values or IDs, there is, of course, no option for a bar chart, and all of the identified metabolites on the maps are coloured yellow.

## DISCUSSION

The functionality of *Pathos* overlaps to a certain extent with that of *MassTRIX*,^17^ but some key novel features are built into *Pathos*. The focus in *MassTRIX* is the identification of potential metabolites from *m/z* data, and it is appropriate that the colour-coding of the pathway maps indicates the relationship of the occurrence of the metabolite in the organism and in the experimental data. The focus of *Pathos*, in contrast, is presentation of relative quantification of experimental results, and was designed in the context of an increasing tendency to pre-process the mass spectrometric data to produce input files with metabolites, the identities of which are already known. Hence, the colour-coding of the metabolic intermediates – the simplest but most effective visualization technique available for pathway maps – is employed to inform the user of metabolites which have undergone quantitative changes, the details of which are instantly accessible by interacting with the map. A more minor difference – although of practical relevance – is that *MassTRIX* provides an extremely comprehensive analysis of an *m/z* dataset, but this typically takes many hours to produce. *Pathos* is designed to be more immediate by providing minimal essential output initially, while allowing users to select subsequently which maps or column charts they wish to generate.

There are some other web applications, such as *MetaboAnalyst*^26^ and *metaP-server*,^27^ that focus on experimental comparisons analyzed by mass spectrometry. These have a strong emphasis on statistical analysis of results, in contrast to *Pathos*, which is essentially a presentation tool and performs no statistical analysis, merely displaying in the column charts any pre-determined standard deviations included in the user's input file.

## CONCLUSIONS

The web application *Pathos* satisfies a need at the visualization point of the pipeline for analyzing metabolomics data. We have found that biological scientists appreciate its simple interface, its speed of response and the familiar format in which their results are imposed on metabolic pathway maps. There is scope for further improvement of the maps – the delay in their generation through the *KEGG* web service being a notable inconvenience, and the maps could be made richer in certain respects. We hope to address these points in the future, for example through integrating the generation of the annotated pathway maps into the *Pathos* application itself.
