# Supplementary material for: Pathos: A web facility that uses metabolic maps to display experimental changes in metabolites identified by mass spectrometry
Source: Rapid Commun Mass Spectrom. 2011 Oct 17;25(22):3422–6. doi: 10.1002/rcm.5245 (PMC3509215; doi:10.1002/rcm.5245)

**SUPPORTING INFORMATION**

**Pathos: A web facility that uses metabolic maps to display experimental changes in metabolites identified by mass spectrometry**

**David P. Leader^1^*, Karl Burgess^2^, Darren Creek^3,4^ and Michael P. Barrett^3^**

^1^School of Life Sciences, College of Medical, Veterinary and Life Sciences, University of Glasgow, Glasgow G12 8QQ, UK

^2^Functional Genomics and Systems Medicine, College of Medical, Veterinary and Life Sciences, Joseph Black Building, University of Glasgow, Glasgow G12 8QQ, UK

^3^Wellcome Trust Centre for Molecular Parasitology, Institute of Infection, Immunity and Inflammation, College of Medical, Veterinary and Life Sciences, University of Glasgow, Glasgow G12 8TA, UK

^4^Department of Biochemistry and Molecular Biology, Bio21 Molecular Science and Biotechnology Institute, University of Melbourne, Flemington Rd, Parkville, Victoria 3010, Australia

**Correspondence to*: D. P. Leader, Joseph Black Building, University of Glasgow, Glasgow G12 8QQ, UK.

E-mail: david.leader@glasgow.ac.uk

**Figure S1.** Schema of database underlying the Pathos web facility. (The 'Compound' and 'Reaction' tables – and the 'ReactionOfMap' and 'ReactionOfMetabolite' tables – are not currently used in any SQL queries from the web application.)

**
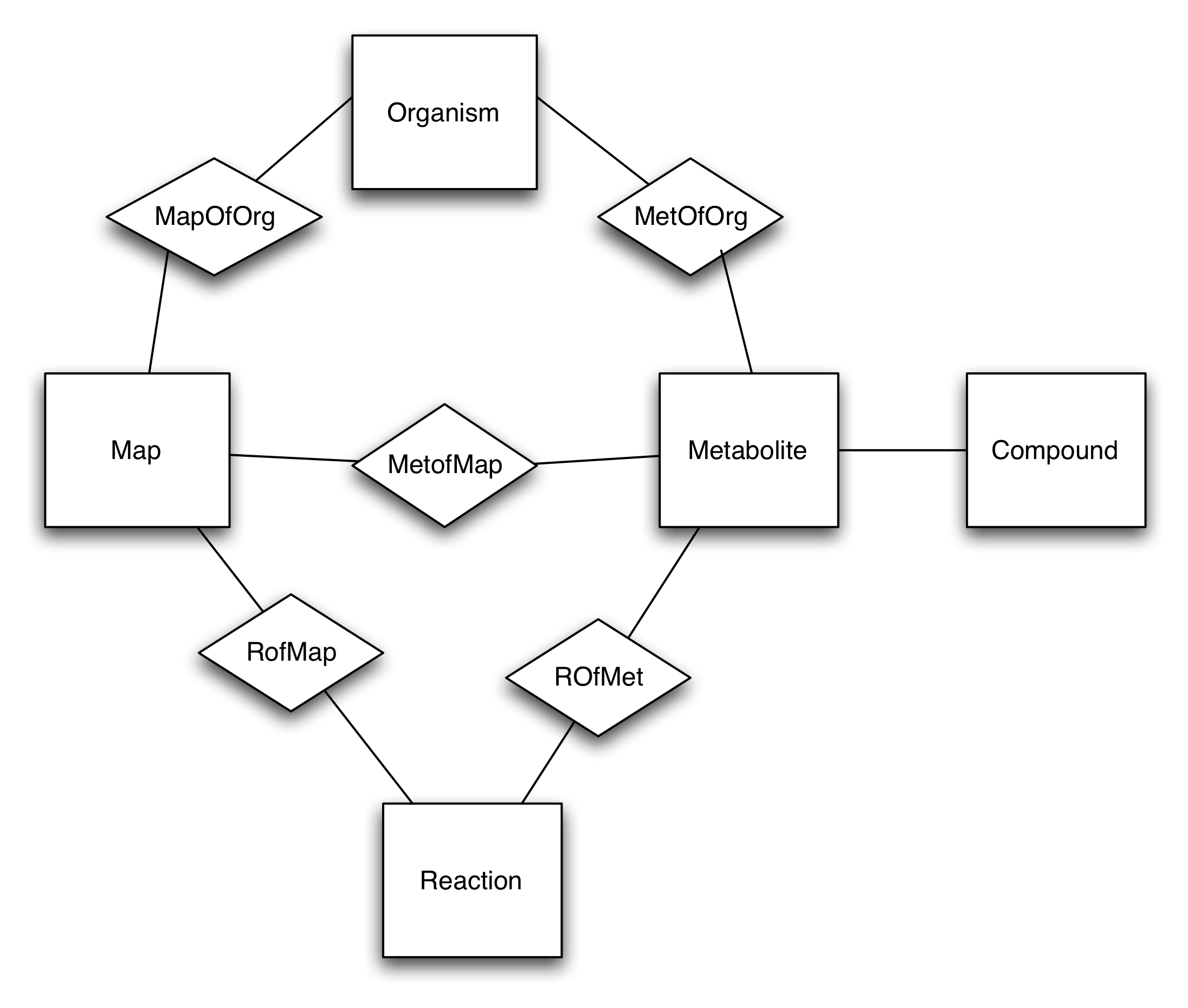
**

**Figure S2.** Tables in the database underlying the Pathos web application. (The 'Compound' and 'Reaction' tables – and the 'ReactionOfMap' and 'ReactionOfMetabolite' tables – are not currently used in any SQL queries from the web application.)

Map

+---------+--------------+------+-----+---------+-------+

| Field | Type | Null | Key | Default | Extra |

+---------+--------------+------+-----+---------+-------+

| MapID | varchar(8) | | PRI | | |

| MapName | varchar(250) | | | | |

+---------+--------------+------+-----+---------+-------+

MapOfOrg

+------------+------------+------+-----+---------+-------+

| Field | Type | Null | Key | Default | Extra |

+------------+------------+------+-----+---------+-------+

| MapID | varchar(8) | | PRI | | |

| OrganismID | char(3) | | PRI | | |

+------------+------------+------+-----+---------+-------+

Metabolite

+--------------+-------------+------+-----+---------+-------+

| Field | Type | Null | Key | Default | Extra |

+--------------+-------------+------+-----+---------+-------+

| CompoundID | varchar(6) | | PRI | | |

| CompoundName | text | | | | |

| Formula | varchar(25) | | | | |

| FormDegen | int(11) | | | 0 | |

| MIM | double | | | 0 | |

| InChI | text | | | | |

| MetacycUID | varchar(50) | YES | | NULL | |

+--------------+-------------+------+-----+---------+-------+

MetaboliteOfMap

+-------------+-------------+------+-----+---------+-------+

| Field | Type | Null | Key | Default | Extra |

+-------------+-------------+------+-----+---------+-------+

| MapID | varchar(8) | | PRI | | |

| CompoundID | varchar(6) | | PRI | | |

| GlyphShape | varchar(20) | | | none | |

| GlyphX | int(4) | | PRI | 0 | |

| GlyphY | int(4) | | PRI | 0 | |

| GlyphWidth | int(3) | | | 0 | |

| GlyphHeight | int(3) | | | 0 | |

+-------------+-------------+------+-----+---------+-------+

MetaboliteOfOrg

+------------+------------+------+-----+---------+-------+

| Field | Type | Null | Key | Default | Extra |

+------------+------------+------+-----+---------+-------+

| OrganismID | char(3) | | PRI | | |

| CompoundID | varchar(6) | | PRI | | |

+------------+------------+------+-----+---------+-------+

Organism

+--------------+-------------+------+-----+---------+-------+

| Field | Type | Null | Key | Default | Extra |

+--------------+-------------+------+-----+---------+-------+

| OrganismID | char(3) | | PRI | | |

| OrganismName | varchar(40) | | | | |

+--------------+-------------+------+-----+---------+-------+

Compound

+--------------+-------------+------+-----+---------+-------+

| Field | Type | Null | Key | Default | Extra |

+--------------+-------------+------+-----+---------+-------+

| CompoundID | varchar(6) | | | | |

| CompoundName | text | | | | |

| Formula | varchar(25) | | | | |

| MIM | double | | | 0 | |

| InChI | text | | | | |

| MetacycUID | varchar(50) | YES | | NULL | |

+--------------+-------------+------+-----+---------+-------+

Reaction

+--------------+------------+------+-----+---------+-------+

| Field | Type | Null | Key | Default | Extra |

+--------------+------------+------+-----+---------+-------+

| ReactionID | varchar(6) | | PRI | | |

| ReactionName | text | | | | |

+--------------+------------+------+-----+---------+-------+

ReactionOfMap

+------------+------------+------+-----+---------+-------+

| Field | Type | Null | Key | Default | Extra |

+------------+------------+------+-----+---------+-------+

| ReactionID | varchar(6) | | PRI | | |

| MapID | varchar(8) | | PRI | | |

+------------+------------+------+-----+---------+-------+

ReactionOfMetabolite

+------------+------------+------+-----+---------+-------+

| Field | Type | Null | Key | Default | Extra |

+------------+------------+------+-----+---------+-------+

| ReactionID | varchar(6) | | PRI | | |

| CompoundID | varchar(6) | | PRI | | |

+------------+------------+------+-----+---------+-------+

**Figure S3.** Map Metabolites in the Kegg Database with masses overlapping at a tolerance of 2 ppm.

| C9H12O4 | 3-Methoxy-4-hydroxyphenylethyleneglycol | 184.07356 | C5H15NO4P | Choline phosphate | 184.073872 |
| --- | --- | --- | --- | --- | --- |
|  | 2-Hydroxy-6-oxo-7-methylocta-2,4-dienoate |  |  |  |  |
|  | cis-3-(Carboxy-ethyl)-3,5-cyclo-hexadiene-1,2-diol |  |  |  |  |
| C15H11O6 | Cyanidin | 287.055565 | C11H14NO6P | Indoleglycerol phosphate | 287.055877 |
| C15H15BrO2 | Monobromobisphenol A | 306.025541 | C9H11N2O8P | 2',3'-Cyclic UMP | 306.025306 |
| C20H15NO5 | 10-Hydroxydihydrosanguinarine | 349.095024 | C12H19N3O7S | S-(Formylmethyl)glutathione | 349.094374 |
| C20H15NO6 | 6-Methylpretetramide | 365.089939 | C12H19N3O8S | 2-S-Glutathionyl acetate | 365.089289 |
| C21H17NO6 | 12-Hydroxydihydrochelirubine | 379.105589 | C13H21N3O8S | (R)-S-Lactoylglutathione | 379.104939 |
| C12H19N4O7P2S | Thiamin diphosphate | 425.044974 | C14H19NO10S2 | Sinalbin | 425.045043 |
| C52H76O12 | Zeaxanthin diglucoside | 892.53368 | C55H72MgN4O5 | Chlorophyll a | 892.535316 |
|  | (2S,2'S)-Oscillol 2,2'-di(alpha-L-fucoside) |  |  |  |  |

**Figure S4.** Example of pop-up displaying metabolite information. The pop-up is generated by a call to a Perl CGI application, formula.cgi, the structural formula being retrieved from the KEGG database.


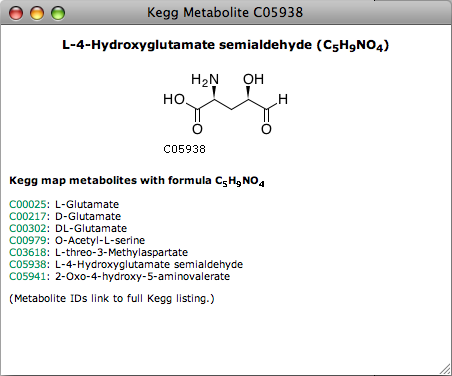

Supplement: Supplementary file 1 [file rcm0025-3422-SD1.doc]
